# Correction to: The 46th Annual Meeting of the Irish Endocrine Society Meeting Programme and Abstracts 2022

**DOI:** 10.1007/s11845-025-03908-5

**Published:** 2025-02-21

**Correction to: Irish Journal of Medical Science (1971 -) (2024) 193 (Suppl 4):S117–S162**

10.1007/s11845-024-03824-0

These abstracts were initially published as non-open access despite being intended for open access publication. This issue has now been resolved.

The original article has been corrected.

